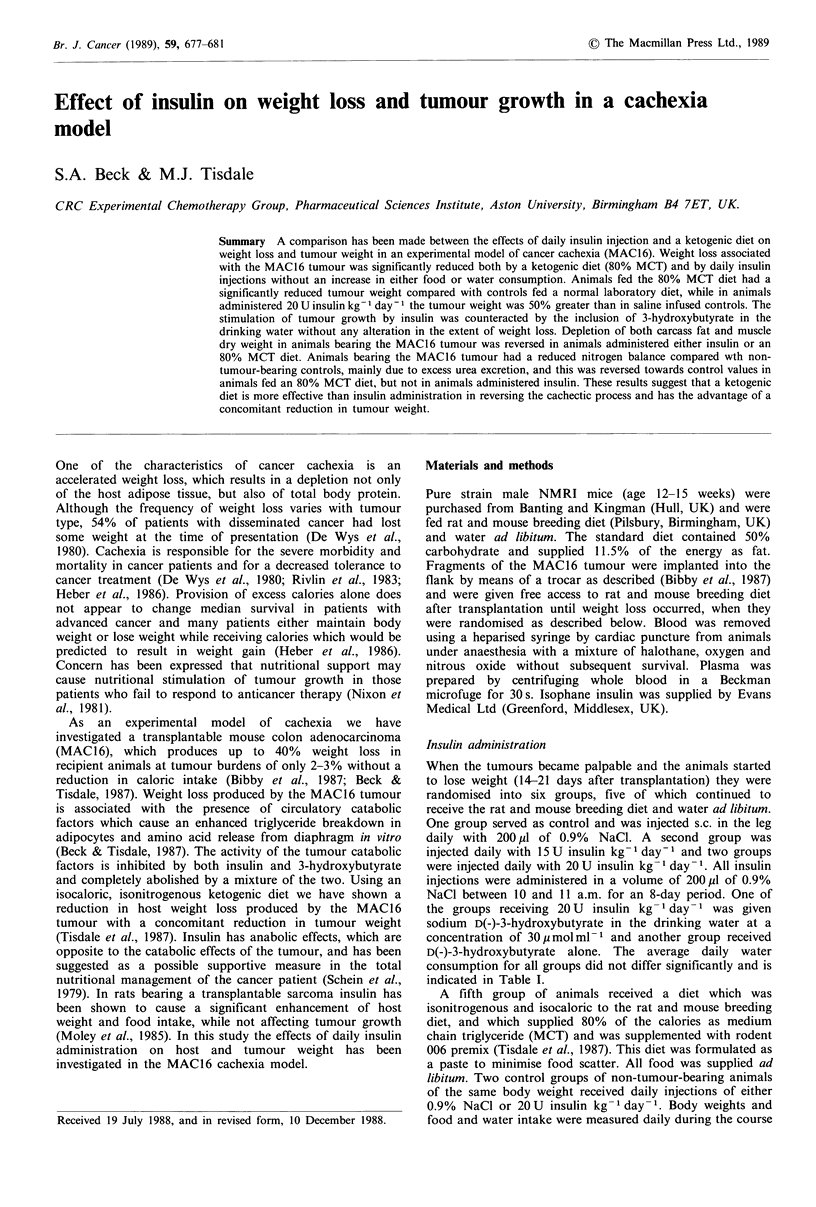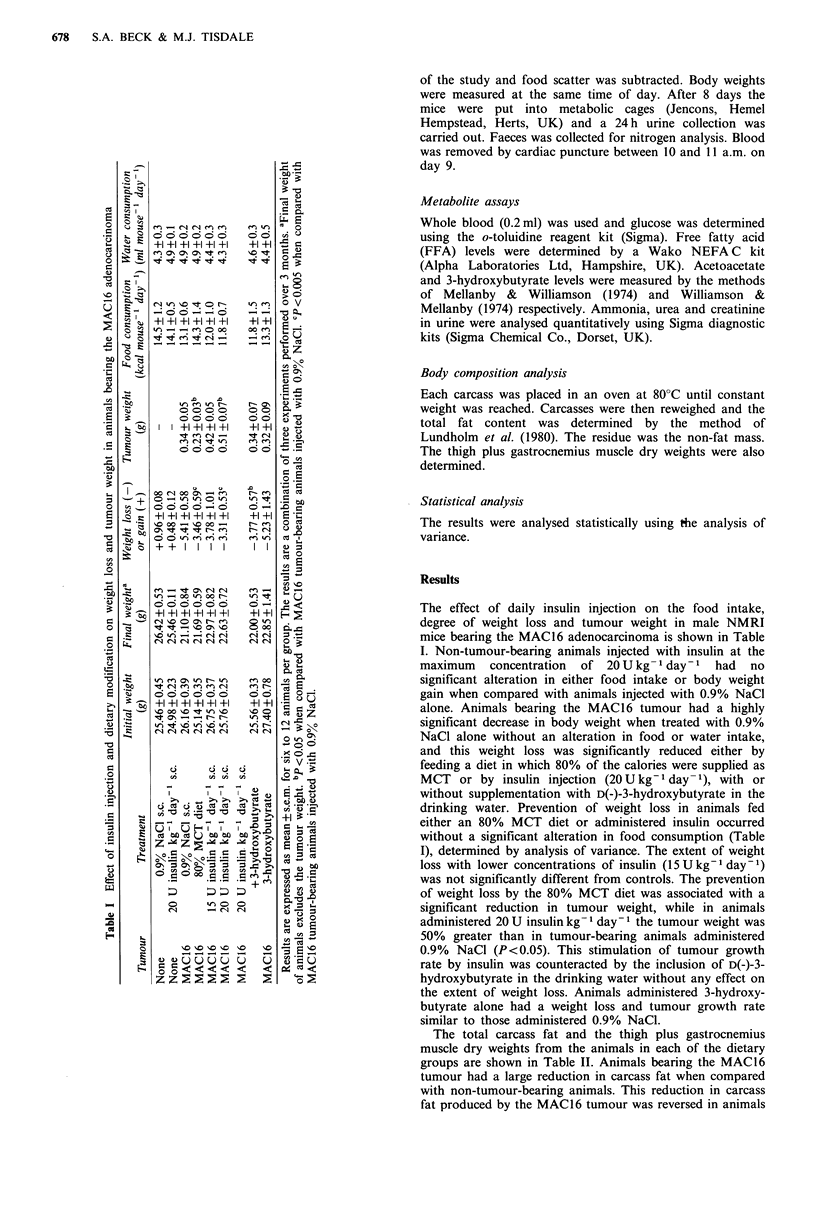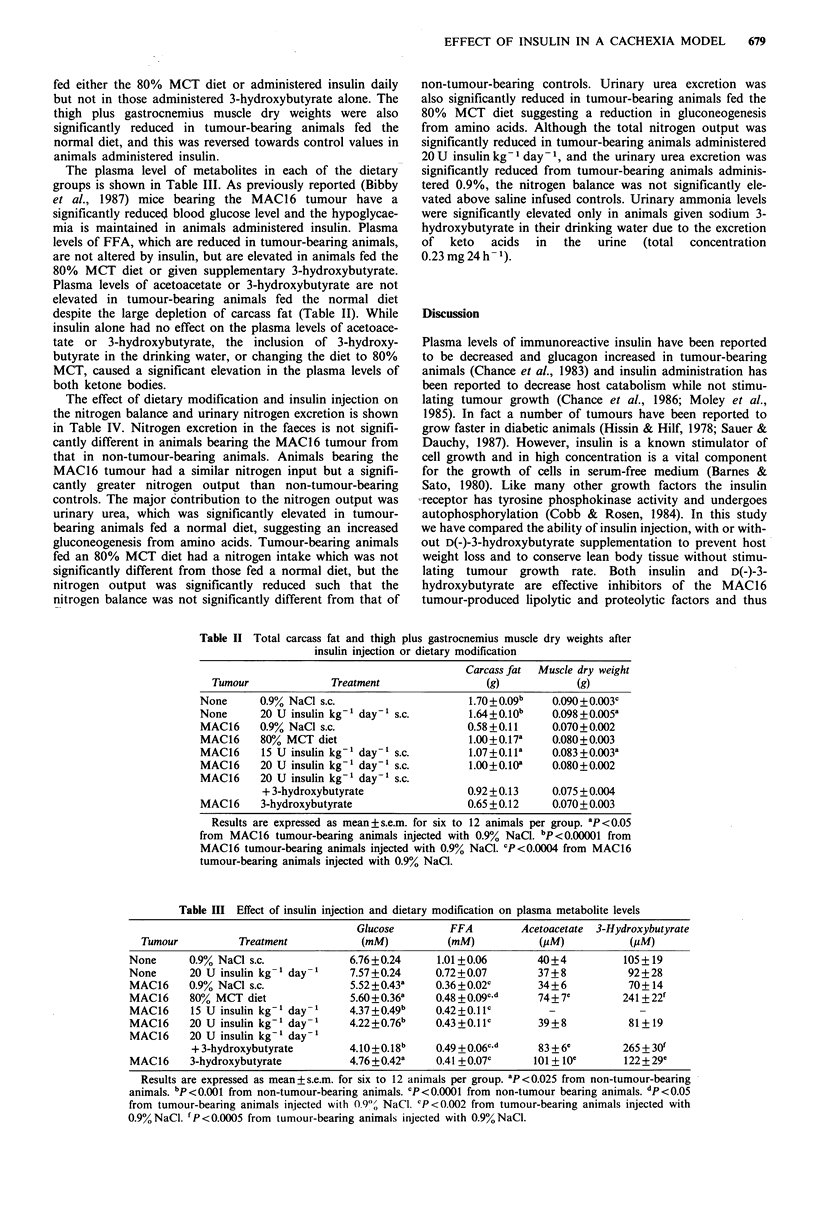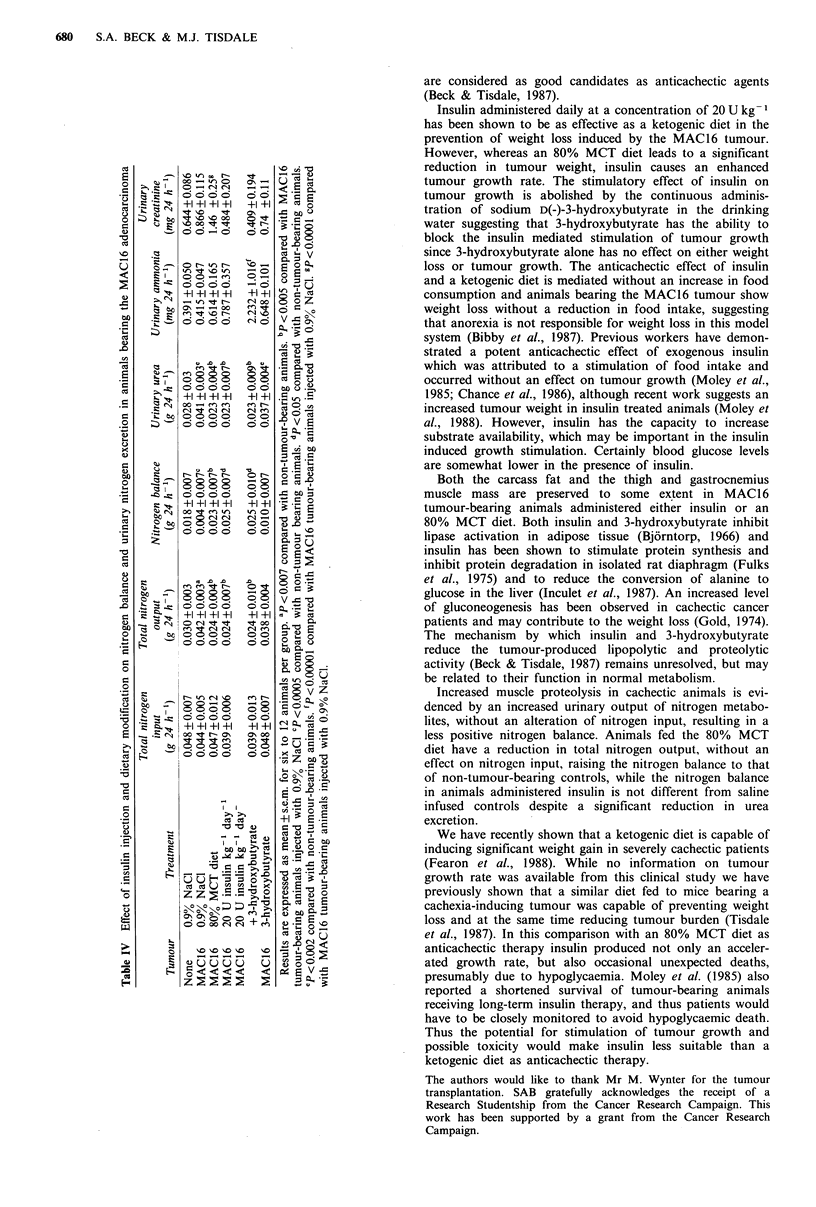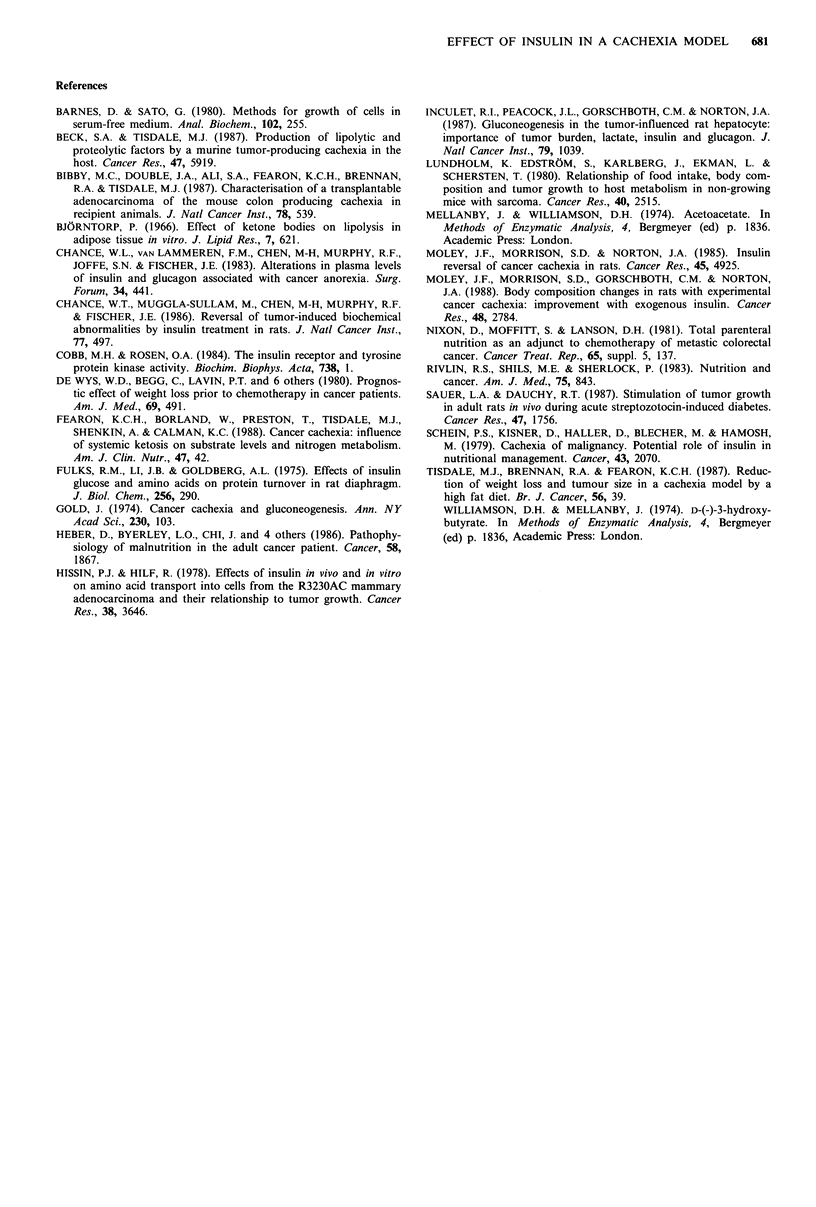# Effect of insulin on weight loss and tumour growth in a cachexia model.

**DOI:** 10.1038/bjc.1989.140

**Published:** 1989-05

**Authors:** S. A. Beck, M. J. Tisdale

**Affiliations:** Pharmaceutical Sciences Institute, Aston University, Birmingham, UK.

## Abstract

A comparison has been made between the effects of daily insulin injection and a ketogenic diet on weight loss and tumour weight in an experimental model of cancer cachexia (MAC16). Weight loss associated with the MAC16 tumour was significantly reduced both by a ketogenic diet (80% MCT) and by daily insulin injections without an increase in either food or water consumption. Animals fed the 80% MCT diet had a significantly reduced tumour weight compared with controls fed a normal laboratory diet, while in animals administered 20 U insulin kg-1 day-1 the tumour weight was 50% greater than in saline infused controls. The stimulation of tumour growth by insulin was counteracted by the inclusion of 3-hydroxybutyrate in the drinking water without any alteration in the extent of weight loss. Depletion of both carcass fat and muscle dry weight in animals bearing the MAC16 tumour was reversed in animals administered either insulin or an 80% MCT diet. Animals bearing the MAC16 tumour had a reduced nitrogen balance compared with non-tumour-bearing controls, mainly due to excess urea excretion, and this was reversed towards control values in animals fed an 80% MCT diet, but not in animals administered insulin. These results suggest that a ketogenic diet is more effective than insulin administration in reversing the cachectic process and has the advantage of a concomitant reduction in tumour weight.


Br. J. Cancer (1989), 59, 677-681 © The Macmillan Press Ltd., 1989

### Effect of insulin on weight loss and tumour growth in a cachexia model

S.A. Beck & M.J. Tisdale

*CRC Experimental Chemotherapy Group, Pharmaceutical Sciences Institute, Aston University, Birmingham B4 7ET, UK.*

**Summary** A comparison has been made between the effects of daily insulin injection and a ketogenic diet on weight loss and tumour weight in an experimental model of cancer cachexia (MAC16). Weight loss associated with the MAC16 tumour was significantly reduced both by a ketogenic diet (80% MCT) and by daily insulin injections without an increase in either food or water consumption. Animals fed the 80% MCT diet had a significantly reduced tumour weight compared with controls fed a normal laboratory diet, while in animals administered 20 U insulin $kg^{-1}$ $day^{-1}$ the tumour weight was 50% greater than in saline infused controls. The stimulation of tumour growth by insulin was counteracted by the inclusion of 3-hydroxybutyrate in the drinking water without any alteration in the extent of weight loss. Depletion of both carcass fat and muscle dry weight in animals bearing the MAC16 tumour was reversed in animals administered either insulin or an 80% MCT diet. Animals bearing the MAC16 tumour had a reduced nitrogen balance compared wth non-tumour-bearing controls, mainly due to excess urea excretion, and this was reversed towards control values in animals fed an 80% MCT diet, but not in animals administered insulin. These results suggest that a ketogenic diet is more effective than insulin administration in reversing the cachectic process and has the advantage of a concomitant reduction in tumour weight.

One of the characteristics of cancer cachexia is an accelerated weight loss, which results in a depletion not only of the host adipose tissue, but also of total body protein. Although the frequency of weight loss varies with tumour type, 54% of patients with disseminated cancer had lost some weight at the time of presentation (De Wys *et al.*, 1980). Cachexia is responsible for the severe morbidity and mortality in cancer patients and for a decreased tolerance to cancer treatment (De Wys *et al.*, 1980; Rivlin *et al.*, 1983; Heber *et al.*, 1986). Provision of excess calories alone does not appear to change median survival in patients with advanced cancer and many patients either maintain body weight or lose weight while receiving calories which would be predicted to result in weight gain (Heber *et al.*, 1986). Concern has been expressed that nutritional support may cause nutritional stimulation of tumour growth in those patients who fail to respond to anticancer therapy (Nixon *et al.*, 1981).

As an experimental model of cachexia we have investigated a transplantable mouse colon adenocarcinoma (MAC16), which produces up to 40% weight loss in recipient animals at tumour burdens of only 2-3% without a reduction in caloric intake (Bibby *et al.*, 1987; Beck & Tisdale, 1987). Weight loss produced by the MAC16 tumour is associated with the presence of circulatory catabolic factors which cause an enhanced triglyceride breakdown in adipocytes and amino acid release from diaphragm *in vitro* (Beck & Tisdale, 1987). The activity of the tumour catabolic factors is inhibited by both insulin and 3-hydroxybutyrate and completely abolished by a mixture of the two. Using an isocaloric, isonitrogenous ketogenic diet we have shown a reduction in host weight loss produced by the MAC16 tumour with a concomitant reduction in tumour weight (Tisdale *et al.*, 1987). Insulin has anabolic effects, which are opposite to the catabolic effects of the tumour, and has been suggested as a possible supportive measure in the total nutritional management of the cancer patient (Schein *et al.*, 1979). In rats bearing a transplantable sarcoma insulin has been shown to cause a significant enhancement of host weight and food intake, while not affecting tumour growth (Moley *et al.*, 1985). In this study the effects of daily insulin administration on host and tumour weight has been investigated in the MAC16 cachexia model.

Received 19 July 1988, and in revised form, 10 December 1988.

### Materials and methods

Pure strain male NMRI mice (age 12-15 weeks) were purchased from Banting and Kingman (Hull, UK) and were fed rat and mouse breeding diet (Pilsbury, Birmingham, UK) and water *ad libitum*. The standard diet contained 50% carbohydrate and supplied 11.5% of the energy as fat. Fragments of the MAC16 tumour were implanted into the flank by means of a trocar as described (Bibby *et al.*, 1987) and were given free access to rat and mouse breeding diet after transplantation until weight loss occurred, when they were randomised as described below. Blood was removed using a heparised syringe by cardiac puncture from animals under anaesthesia with a mixture of halothane, oxygen and nitrous oxide without subsequent survival. Plasma was prepared by centrifuging whole blood in a Beckman microfuge for 30 s. Isophane insulin was supplied by Evans Medical Ltd (Greenford, Middlesex, UK).

### *Insulin administration*

When the tumours became palpable and the animals started to lose weight (14-21 days after transplantation) they were randomised into six groups, five of which continued to receive the rat and mouse breeding diet and water *ad libitum*. One group served as control and was injected s.c. in the leg daily with 200 $\mu$l of 0.9% NaCl. A second group was injected daily with 15 U insulin $kg^{-1}$ $day^{-1}$ and two groups were injected daily with 20 U insulin $kg^{-1}$ $day^{-1}$. All insulin injections were administered in a volume of 200 $\mu$l of 0.9% NaCl between 10 and 11 a.m. for an 8-day period. One of the groups receiving 20 U insulin $kg^{-1}$ $day^{-1}$ was given sodium D(-)-3-hydroxybutyrate in the drinking water at a concentration of 30 $\mu$mol $ml^{-1}$ and another group received D(-)-3-hydroxybutyrate alone. The average daily water consumption for all groups did not differ significantly and is indicated in Table I.

A fifth group of animals received a diet which was isonitrogenous and isocaloric to the rat and mouse breeding diet, and which supplied 80% of the calories as medium chain triglyceride (MCT) and was supplemented with rodent 006 premix (Tisdale *et al.*, 1987). This diet was formulated as a paste to minimise food scatter. All food was supplied *ad libitum*. Two control groups of non-tumour-bearing animals of the same body weight received daily injections of either 0.9% NaCl or 20 U insulin $kg^{-1}$ $day^{-1}$. Body weights and food and water intake were measured daily during the course of the study and food scatter was subtracted. Body weights were measured at the same time of day. After 8 days the mice were put into metabolic cages (Jencons, Hemel Hempstead, Herts, UK) and a 24 h urine collection was carried out. Faeces was collected for nitrogen analysis. Blood was removed by cardiac puncture between 10 and 11 a.m. on day 9.

### *Metabolite assays*

Whole blood (0.2 ml) was used and glucose was determined using the *o*-toluidine reagent kit (Sigma). Free fatty acid (FFA) levels were determined by a Wako NEFA C kit (Alpha Laboratories Ltd, Hampshire, UK). Acetoacetate and 3-hydroxybutyrate levels were measured by the methods of Mellanby & Williamson (1974) and Williamson & Mellanby (1974) respectively. Ammonia, urea and creatinine in urine were analysed quantitatively using Sigma diagnostic kits (Sigma Chemical Co., Dorset, UK).

### *Body composition analysis*

Each carcass was placed in an oven at 80°C until constant weight was reached. Carcasses were then reweighed and the total fat content was determined by the method of Lundholm *et al.* (1980). The residue was the non-fat mass. The thigh plus gastrocnemius muscle dry weights were also determined.

### *Statistical analysis*

The results were analysed statistically using the analysis of variance.

### Results

The effect of daily insulin injection on the food intake, degree of weight loss and tumour weight in male NMRI mice bearing the MAC16 adenocarcinoma is shown in Table I. Non-tumour-bearing animals injected with insulin at the maximum concentration of 20 U kg$^{-1}$ day$^{-1}$ had no significant alteration in either food intake or body weight gain when compared with animals injected with 0.9% NaCl alone. Animals bearing the MAC16 tumour had a highly significant decrease in body weight when treated with 0.9% NaCl alone without an alteration in food or water intake, and this weight loss was significantly reduced either by feeding a diet in which 80% of the calories were supplied as MCT or by insulin injection (20 U kg$^{-1}$ day$^{-1}$), with or without supplementation with D(-)-3-hydroxybutyrate in the drinking water. Prevention of weight loss in animals fed either an 80% MCT diet or administered insulin occurred without a significant alteration in food consumption (Table I), determined by analysis of variance. The extent of weight loss with lower concentrations of insulin (15 U kg$^{-1}$ day$^{-1}$) was not significantly different from controls. The prevention of weight loss by the 80% MCT diet was associated with a significant reduction in tumour weight, while in animals administered 20 U insulin kg$^{-1}$ day$^{-1}$ the tumour weight was 50% greater than in tumour-bearing animals administered 0.9% NaCl ($P<0.05$). This stimulation of tumour growth rate by insulin was counteracted by the inclusion of D(-)-3-hydroxybutyrate in the drinking water without any effect on the extent of weight loss. Animals administered 3-hydroxybutyrate alone had a weight loss and tumour growth rate similar to those administered 0.9% NaCl.

The total carcass fat and the thigh plus gastrocnemius muscle dry weights from the animals in each of the dietary groups are shown in Table II. Animals bearing the MAC16 tumour had a large reduction in carcass fat when compared with non-tumour-bearing animals. This reduction in carcass fat produced by the MAC16 tumour was reversed in animals fed either the 80% MCT diet or administered insulin daily but not in those administered 3-hydroxybutyrate alone. The thigh plus gastrocnemius muscle dry weights were also significantly reduced in tumour-bearing animals fed the normal diet, and this was reversed towards control values in animals administered insulin.

The plasma level of metabolites in each of the dietary groups is shown in Table III. As previously reported (Bibby *et al.*, 1987) mice bearing the MAC16 tumour have a significantly reduced blood glucose level and the hypoglycaemia is maintained in animals administered insulin. Plasma levels of FFA, which are reduced in tumour-bearing animals, are not altered by insulin, but are elevated in animals fed the 80% MCT diet or given supplementary 3-hydroxybutyrate. Plasma levels of acetoacetate or 3-hydroxybutyrate are not elevated in tumour-bearing animals fed the normal diet despite the large depletion of carcass fat (Table II). While insulin alone had no effect on the plasma levels of acetoacetate or 3-hydroxybutyrate, the inclusion of 3-hydroxybutyrate in the drinking water, or changing the diet to 80% MCT, caused a significant elevation in the plasma levels of both ketone bodies.

The effect of dietary modification and insulin injection on the nitrogen balance and urinary nitrogen excretion is shown in Table IV. Nitrogen excretion in the faeces is not significantly different in animals bearing the MAC16 tumour from that in non-tumour-bearing animals. Animals bearing the MAC16 tumour had a similar nitrogen input but a significantly greater nitrogen output than non-tumour-bearing controls. The major contribution to the nitrogen output was urinary urea, which was significantly elevated in tumour-bearing animals fed a normal diet, suggesting an increased gluconeogenesis from amino acids. Tumour-bearing animals fed an 80% MCT diet had a nitrogen intake which was not significantly different from those fed a normal diet, but the nitrogen output was significantly reduced such that the nitrogen balance was not significantly different from that of non-tumour-bearing controls. Urinary urea excretion was also significantly reduced in tumour-bearing animals fed the 80% MCT diet suggesting a reduction in gluconeogenesis from amino acids. Although the total nitrogen output was significantly reduced in tumour-bearing animals administered 20 U insulin $kg^{-1}$ $day^{-1}$, and the urinary urea excretion was significantly reduced from tumour-bearing animals administered 0.9%, the nitrogen balance was not significantly elevated above saline infused controls. Urinary ammonia levels were significantly elevated only in animals given sodium 3-hydroxybutyrate in their drinking water due to the excretion of keto acids in the urine (total concentration 0.23 mg 24 $h^{-1}$).

### Discussion

Plasma levels of immunoreactive insulin have been reported to be decreased and glucagon increased in tumour-bearing animals (Chance *et al.*, 1983) and insulin administration has been reported to decrease host catabolism while not stimulating tumour growth (Chance *et al.*, 1986; Moley *et al.*, 1985). In fact a number of tumours have been reported to grow faster in diabetic animals (Hissin & Hilf, 1978; Sauer & Dauchy, 1987). However, insulin is a known stimulator of cell growth and in high concentration is a vital component for the growth of cells in serum-free medium (Barnes & Sato, 1980). Like many other growth factors the insulin receptor has tyrosine phosphokinase activity and undergoes autophosphorylation (Cobb & Rosen, 1984). In this study we have compared the ability of insulin injection, with or without D(-)-3-hydroxybutyrate supplementation to prevent host weight loss and to conserve lean body tissue without stimulating tumour growth rate. Both insulin and D(-)-3-hydroxybutyrate are effective inhibitors of the MAC16 tumour-produced lipolytic and proteolytic factors and thus

**Table II** Total carcass fat and thigh plus gastrocnemius muscle dry weights after insulin injection or dietary modification

| *Tumour* | *Treatment* | *Carcass fat (g)* | *Muscle dry weight (g)* |
|---|---|---|---|
| None | 0.9% NaCl s.c. | 1.70±0.09[b] | 0.090±0.003[c] |
| None | 20 U insulin $kg^{-1}$ $day^{-1}$ s.c. | 1.64±0.10[b] | 0.098±0.005[a] |
| MAC16 | 0.9% NaCl s.c. | 0.58±0.11 | 0.070±0.002 |
| MAC16 | 80% MCT diet | 1.00±0.17[a] | 0.080±0.003 |
| MAC16 | 15 U insulin $kg^{-1}$ $day^{-1}$ s.c. | 1.07±0.11[a] | 0.083±0.003[a] |
| MAC16 | 20 U insulin $kg^{-1}$ $day^{-1}$ s.c. | 1.00±0.10[a] | 0.080±0.002 |
| MAC16 | 20 U insulin $kg^{-1}$ $day^{-1}$ s.c. + 3-hydroxybutyrate | 0.92±0.13 | 0.075±0.004 |
| MAC16 | 3-hydroxybutyrate | 0.65±0.12 | 0.070±0.003 |

Results are expressed as mean±s.e.m. for six to 12 animals per group. [a]$P<0.05$ from MAC16 tumour-bearing animals injected with 0.9% NaCl. [b]$P<0.00001$ from MAC16 tumour-bearing animals injected with 0.9% NaCl. [c]$P<0.0004$ from MAC16 tumour-bearing animals injected with 0.9% NaCl.

**Table III** Effect of insulin injection and dietary modification on plasma metabolite levels

| *Tumour* | *Treatment* | *Glucose (mM)* | *FFA (mM)* | *Acetoacetate (μM)* | *3-Hydroxybutyrate (μM)* |
|---|---|---|---|---|---|
| None | 0.9% NaCl s.c. | 6.76±0.24 | 1.01±0.06 | 40±4 | 105±19 |
| None | 20 U insulin $kg^{-1}$ $day^{-1}$ | 7.57±0.24 | 0.72±0.07 | 37±8 | 92±28 |
| MAC16 | 0.9% NaCl s.c. | 5.52±0.43[a] | 0.36±0.02[c] | 34±6 | 70±14 |
| MAC16 | 80% MCT diet | 5.60±0.36[a] | 0.48±0.09[c,d] | 74±7[e] | 241±22[f] |
| MAC16 | 15 U insulin $kg^{-1}$ $day^{-1}$ | 4.37±0.49[b] | 0.42±0.11[c] | – | – |
| MAC16 | 20 U insulin $kg^{-1}$ $day^{-1}$ | 4.22±0.76[b] | 0.43±0.11[c] | 39±8 | 81±19 |
| MAC16 | 20 U insulin $kg^{-1}$ $day^{-1}$ + 3-hydroxybutyrate | 4.10±0.18[b] | 0.49±0.06[c,d] | 83±6[e] | 265±30[f] |
| MAC16 | 3-hydroxybutyrate | 4.76±0.42[a] | 0.41±0.07[c] | 101±10[e] | 122±29[e] |

Results are expressed as mean±s.e.m. for six to 12 animals per group. [a]$P<0.025$ from non-tumour-bearing animals. [b]$P<0.001$ from non-tumour-bearing animals. [c]$P<0.0001$ from non-tumour bearing animals. [d]$P<0.05$ from tumour-bearing animals injected with 0.9% NaCl. [e]$P<0.002$ from tumour-bearing animals injected with 0.9% NaCl. [f]$P<0.0005$ from tumour-bearing animals injected with 0.9% NaCl.

are considered as good candidates as anticachectic agents (Beck & Tisdale, 1987).

Insulin administered daily at a concentration of 20 U $kg^{-1}$ has been shown to be as effective as a ketogenic diet in the prevention of weight loss induced by the MAC16 tumour. However, whereas an 80% MCT diet leads to a significant reduction in tumour weight, insulin causes an enhanced tumour growth rate. The stimulatory effect of insulin on tumour growth is abolished by the continuous administration of sodium D(-)-3-hydroxybutyrate in the drinking water suggesting that 3-hydroxybutyrate has the ability to block the insulin mediated stimulation of tumour growth since 3-hydroxybutyrate alone has no effect on either weight loss or tumour growth. The anticachectic effect of insulin and a ketogenic diet is mediated without an increase in food consumption and animals bearing the MAC16 tumour show weight loss without a reduction in food intake, suggesting that anorexia is not responsible for weight loss in this model system (Bibby *et al.*, 1987). Previous workers have demonstrated a potent anticachectic effect of exogenous insulin which was attributed to a stimulation of food intake and occurred without an effect on tumour growth (Moley *et al.*, 1985; Chance *et al.*, 1986), although recent work suggests an increased tumour weight in insulin treated animals (Moley *et al.*, 1988). However, insulin has the capacity to increase substrate availability, which may be important in the insulin induced growth stimulation. Certainly blood glucose levels are somewhat lower in the presence of insulin.

Both the carcass fat and the thigh and gastrocnemius muscle mass are preserved to some extent in MAC16 tumour-bearing animals administered either insulin or an 80% MCT diet. Both insulin and 3-hydroxybutyrate inhibit lipase activation in adipose tissue (Björntorp, 1966) and insulin has been shown to stimulate protein synthesis and inhibit protein degradation in isolated rat diaphragm (Fulks *et al.*, 1975) and to reduce the conversion of alanine to glucose in the liver (Inculet *et al.*, 1987). An increased level of gluconeogenesis has been observed in cachectic cancer patients and may contribute to the weight loss (Gold, 1974). The mechanism by which insulin and 3-hydroxybutyrate reduce the tumour-produced lipopolytic and proteolytic activity (Beck & Tisdale, 1987) remains unresolved, but may be related to their function in normal metabolism.

Increased muscle proteolysis in cachectic animals is evidenced by an increased urinary output of nitrogen metabolites, without an alteration of nitrogen input, resulting in a less positive nitrogen balance. Animals fed the 80% MCT diet have a reduction in total nitrogen output, without an effect on nitrogen input, raising the nitrogen balance to that of non-tumour-bearing controls, while the nitrogen balance in animals administered insulin is not different from saline infused controls despite a significant reduction in urea excretion.

We have recently shown that a ketogenic diet is capable of inducing significant weight gain in severely cachectic patients (Fearon *et al.*, 1988). While no information on tumour growth rate was available from this clinical study we have previously shown that a similar diet fed to mice bearing a cachexia-inducing tumour was capable of preventing weight loss and at the same time reducing tumour burden (Tisdale *et al.*, 1987). In this comparison with an 80% MCT diet as anticachectic therapy insulin produced not only an accelerated growth rate, but also occasional unexpected deaths, presumably due to hypoglycaemia. Moley *et al.* (1985) also reported a shortened survival of tumour-bearing animals receiving long-term insulin therapy, and thus patients would have to be closely monitored to avoid hypoglycaemic death. Thus the potential for stimulation of tumour growth and possible toxicity would make insulin less suitable than a ketogenic diet as anticachectic therapy.

The authors would like to thank Mr M. Wynter for the tumour transplantation. SAB gratefully acknowledges the receipt of a Research Studentship from the Cancer Research Campaign. This work has been supported by a grant from the Cancer Research Campaign.

### References

BARNES, D. & SATO, G. (1980). Methods for growth of cells in serum-free medium. *Anal. Biochem.*, **102,** 255.

BECK, S.A. & TISDALE, M.J. (1987). Production of lipolytic and proteolytic factors by a murine tumor-producing cachexia in the host. *Cancer Res.*, **47,** 5919.

BIBBY, M.C., DOUBLE, J.A., ALI, S.A., FEARON, K.C.H., BRENNAN, R.A. & TISDALE, M.J. (1987). Characterisation of a transplantable adenocarcinoma of the mouse colon producing cachexia in recipient animals. *J. Natl Cancer Inst.*, **78,** 539.

BJÖRNTORP, P. (1966). Effect of ketone bodies on lipolysis in adipose tissue *in vitro*. *J. Lipid Res.*, **7,** 621.

CHANCE, W.L., VAN LAMMEREN, F.M., CHEN, M-H, MURPHY, R.F., JOFFE, S.N. & FISCHER, J.E. (1983). Alterations in plasma levels of insulin and glucagon associated with cancer anorexia. *Surg. Forum*, **34,** 441.

CHANCE, W.T., MUGGLA-SULLAM, M., CHEN, M-H, MURPHY, R.F. & FISCHER, J.E. (1986). Reversal of tumor-induced biochemical abnormalities by insulin treatment in rats. *J. Natl Cancer Inst.*, **77,** 497.

COBB, M.H. & ROSEN, O.A. (1984). The insulin receptor and tyrosine protein kinase activity. *Biochim. Biophys. Acta*, **738,** 1.

DE WYS, W.D., BEGG, C., LAVIN, P.T. and 6 others (1980). Prognostic effect of weight loss prior to chemotherapy in cancer patients. *Am. J. Med.*, **69,** 491.

FEARON, K.C.H., BORLAND, W., PRESTON, T., TISDALE, M.J., SHENKIN, A. & CALMAN, K.C. (1988). Cancer cachexia: influence of systemic ketosis on substrate levels and nitrogen metabolism. *Am. J. Clin. Nutr.*, **47,** 42.

FULKS, R.M., LI, J.B. & GOLDBERG, A.L. (1975). Effects of insulin glucose and amino acids on protein turnover in rat diaphragm. *J. Biol. Chem.*, **256,** 290.

GOLD, J. (1974). Cancer cachexia and gluconeogenesis. *Ann. NY Acad Sci.*, **230,** 103.

HEBER, D., BYERLEY, L.O., CHI, J. and 4 others (1986). Pathophysiology of malnutrition in the adult cancer patient. *Cancer*, **58,** 1867.

HISSIN, P.J. & HILF, R. (1978). Effects of insulin *in vivo* and *in vitro* on amino acid transport into cells from the R3230AC mammary adenocarcinoma and their relationship to tumor growth. *Cancer Res.*, **38,** 3646.

INCULET, R.I., PEACOCK, J.L., GORSCHBOTH, C.M. & NORTON, J.A. (1987). Gluconeogenesis in the tumor-influenced rat hepatocyte: importance of tumor burden, lactate, insulin and glucagon. *J. Natl Cancer Inst.*, **79,** 1039.

LUNDHOLM, K. EDSTRÖM, S., KARLBERG, J., EKMAN, L. & SCHERSTEN, T. (1980). Relationship of food intake, body composition and tumor growth to host metabolism in non-growing mice with sarcoma. *Cancer Res.*, **40,** 2515.

MELLANBY, J. & WILLIAMSON, D.H. (1974). Acetoacetate. In *Methods of Enzymatic Analysis, 4,* Bergmeyer (ed) p. 1836. Academic Press: London.

MOLEY, J.F., MORRISON, S.D. & NORTON, J.A. (1985). Insulin reversal of cancer cachexia in rats. *Cancer Res.*, **45,** 4925.

MOLEY, J.F., MORRISON, S.D., GORSCHBOTH, C.M. & NORTON, J.A. (1988). Body composition changes in rats with experimental cancer cachexia: improvement with exogenous insulin. *Cancer Res.*, **48,** 2784.

NIXON, D., MOFFITT, S. & LANSON, D.H. (1981). Total parenteral nutrition as an adjunct to chemotherapy of metastic colorectal cancer. *Cancer Treat. Rep.*, **65,** suppl. 5, 137.

RIVLIN, R.S., SHILS, M.E. & SHERLOCK, P. (1983). Nutrition and cancer. *Am. J. Med.*, **75,** 843.

SAUER, L.A. & DAUCHY, R.T. (1987). Stimulation of tumor growth in adult rats *in vivo* during acute streptozotocin-induced diabetes. *Cancer Res.*, **47,** 1756.

SCHEIN, P.S., KISNER, D., HALLER, D., BLECHER, M. & HAMOSH, M. (1979). Cachexia of malignancy. Potential role of insulin in nutritional management. *Cancer*, **43,** 2070.

TISDALE, M.J., BRENNAN, R.A. & FEARON, K.C.H. (1987). Reduction of weight loss and tumour size in a cachexia model by a high fat diet. *Br. J. Cancer*, **56,** 39.

WILLIAMSON, D.H. & MELLANBY, J. (1974). D-(-)-3-hydroxybutyrate. In *Methods of Enzymatic Analysis, 4*, Bergmeyer (ed) p. 1836, Academic Press: London.